# Isolation and whole genome sequencing of an Avian orthoavulavirus 16 from cinereous vulture (*Aegypius monachus*) in South Korea, 2023

**DOI:** 10.3389/fvets.2026.1739779

**Published:** 2026-02-06

**Authors:** Yongwoo Son, Sun-Hak Lee, Dong-Yeop Lee, Hyunjun Cho, Yemoon Son, Daehun Kim, Chang-Seon Song, Dong-Hun Lee

**Affiliations:** 1 Wildlife Health Laboratory, College of Veterinary Medicine, Konkuk University, Seoul, Republic of Korea; 2 Avian Disease Laboratory, College of Veterinary Medicine, Konkuk University, Seoul, Republic of Korea; 3 Wildlife Disease Research Team, National Institute of Wildlife Disease Control and Prevention, Gwangju, Republic of Korea

**Keywords:** Avian orthoavulavirus, cinereous vulture, serotype 16, whole genome sequencing, wild bird

## 1 Introduction

Avian avulaviruses encompass a diverse group of negative-sense, single-stranded RNA viruses within the subfamily *Avulavirinae* (family *Paramyxoviridae*), currently comprising 22 recognized serotypes (Avian paramyxovirus, APMV-1 to APMV-22) that predominantly circulate in wild and domestic avian species worldwide (1, 2). While Avian orthoavulavirus 1 (AOAV-1)—the causative agent of Newcastle disease—remains the most clinically and economically significant due to its potential high virulence in poultry (3), the majority of non-AOAV-1 serotypes, including Avian orthoavulavirus 16 (AOAV-16), are generally considered low-pathogenic or asymptomatic in their natural hosts (4–6).

*Orthoavulavirus upoense*, known as AOAV-16, was first characterized in 2014 from fecal samples of overwintering wild birds in South Korea, marking its initial detection in East Asia (6). Subsequent genomic surveillance identified AOAV-16 in wild waterfowl across Central Asia, including a virus archived in 2006 from Kazakhstan, suggesting a broader distribution along migratory flyways connecting East Asia, Siberia, and other regions of Eurasia (7, 8). AOAV-16 contains a non-segmented, ~15.1-kb negative-sense RNA genome arranged in the canonical avulavirus gene order (3'-nucleoprotein (NP)–phosphoprotein (P)–matrix protein (M)–fusion protein (F)–hemagglutinin–neuraminidase (HN)–large polymerase protein (L)-5') (6). Phylogenetic analyses based on complete genome sequences and NP gene sequences showed that AOAV-16 is genetically closest to class I lentogenic lineages commonly isolated from *Anseriformes*, supporting a possible shared evolutionary origin and ecological association (7, 8).

Despite these surveillance findings, genomic resources for AOAV-16 remain extremely scarce, with only a handful of complete or near-complete genomes available in public databases. This scarcity limits robust inference regarding viral evolution, host range, recombination potential, and risk of spillover into domestic poultry. Moreover, most confirmed AOAV-16 detections have originated from wild waterfowl, underscoring uncertainties surrounding its host range and the potential involvement of non-*Anseriformes* species in viral maintenance and dissemination.

In this study, we report the first coding-complete genome sequence of AOAV-16 obtained from a fecal dropping of a cinereous vulture (*Aegypius monachus*) collected from South Korea in 2023. This detection demonstrates the presence of AOAV-16 genomic RNA beyond aquatic birds, including *Accipitriformes*, a taxonomic group not previously associated with this serotype. The genome sequences and their phylogenetic relationships provide valuable reference data for future APMV surveillance and research.

## 2 Methods

### 2.1 Sample collection and virus identification

A fecal sample was collected from a fallow paddy field near the Geum River, South Korea (GPS coordinates = 36.0455°N, 126.7434°E) on 26 December 2023 by the National Institute of Wildlife Disease Control and Prevention (NIWDC) as part of the national wild bird surveillance program for avian influenza virus in South Korea. The sample was inoculated into 10-day-old embryonated chicken eggs. Allantoic fluid was tested by hemagglutination assay and screened for influenza A virus by quantitative reverse transcription–polymerase chain reaction (qRT-PCR) using Qiagen QuantiTect RT-PCR reagents (Qiagen, Manchester, UK), following a previously described protocol (9). Hemagglutination-positive but influenza-negative allantoic fluid was filtered through a 0.22-μm syringe filter, and RNA was extracted from allantoic fluid using the RNeasy Mini Kit (Qiagen, Germany). The RNA was subjected to semi-nested RT-PCR targeting the paramyxovirus large protein (L) gene (10). The host species was identified by mitochondrial cytochrome oxidase I (COI) DNA barcoding of the fecal sample (11).

### 2.2 Whole genome sequencing

For whole genome sequencing, viral RNA was amplified using sequence-independent single-primer amplification (SISPA) (12), and a sequencing library was prepared using the Illumina DNA Prep kit. Sequencing was performed on a MiniSeq platform (Illumina, USA). Metagenomic classification was performed using the Chan Zuckerberg ID (CZID) (13) to identify viral reads and guide reference sequence selection. Following adapter and SISPA-primer removal with BBDuk (14) and Geneious Prime 2025.2.2 (https://www.geneious.com), quality-filtered reads were assembled using Minimap2 with *Orthoavulavirus upoense* (GenBank accession no. OR270139) as a reference and deposited in GenBank (accession no. PX482736).

### 2.3 Phylogenetic analysis

Complete genome sequences of avulavirus references designated by the International Committee on Taxonomy of Viruses (ICTV) were retrieved from GenBank. Maximum-likelihood (ML) phylogenetic trees were reconstructed using IQ-TREE v3.0.1 under the best-fit substitution model determined by ModelFinder (15). Branch support was assessed with 1,000 replicates of the Shimodaira–Hasegawa approximate likelihood ratio test and ultrafast bootstrap. Trees were visualized using iTOL v7 (16). Each coding DNA sequence (CDS) of AOAV-16 was pairwise compared at the nucleotide level to calculate sequence identity.

To examine genetic diversity within AOAV-16, all available complete genome and F gene sequences were retrieved from GenBank and analyzed using the same ML approach. Temporal phylogenetic analysis of the F gene was performed using BEAST v10.5.0 under the TN93+F+I model, selected by ModelFinder, with an uncorrelated lognormal relaxed clock and a Gaussian Markov random field (GMRF) Bayesian Skyride coalescent prior (17). Five independent Markov chain Monte Carlo (MCMC) runs of 50 million generations each were combined after discarding the first 10% burn-in. Convergence was assessed using Tracer v1.7.2 (http://tree.bio.ed.ac.uk/software/tracer/), and the maximum clade credibility (MCC) tree was generated with TreeAnnotator and visualized using FigTree v1.4.5 (http://tree.bio.ed.ac.uk/software/figtree/).

## 3 Descriptive results

**Figure 1:**
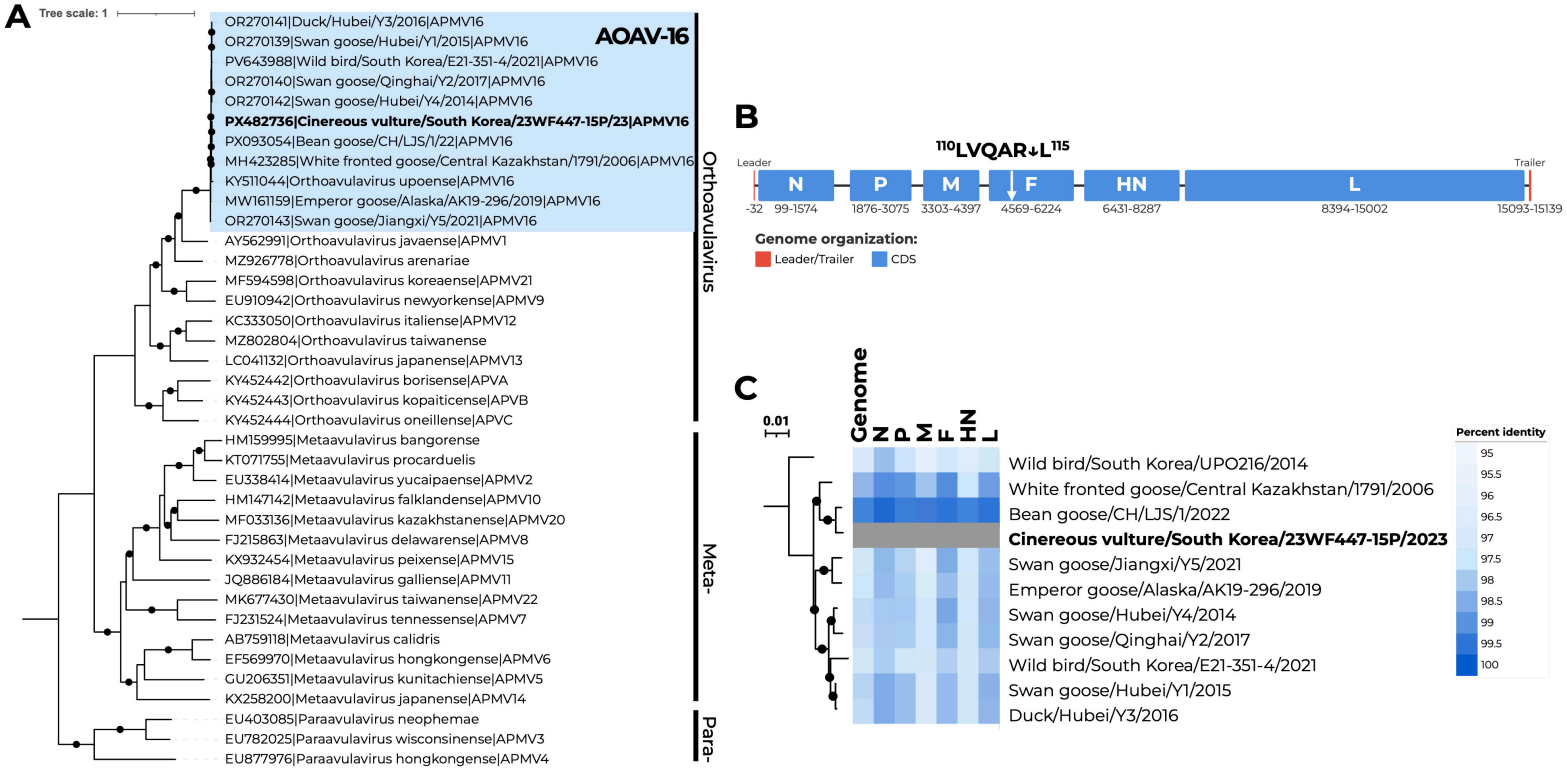
Phylogenetic and genomic characterization of Avian orthoavulavirus 16 (AOAV-16). **(A)** Maximum-likelihood phylogeny of representative avulaviruses inferred using IQ-TREE v3.0.1 under the GTR+F+R5 substitution model. Branches supported by ultrafast bootstrap values of >90% and SH-aLRT values of >70% are marked with black circles. The vulture-derived AOAV-16 sequence generated in this study is shown in bold. The blue box highlights AOAV-16 sequences included in the analysis. **(B)** Genomic organization of the vulture-derived AOAV-16 isolate. Each box represents a coding sequence (CDS) or leader/trailer region, with corresponding nucleotide positions indicated below each CDS. **(C)** Pairwise nucleotide identity among AOAV-16 strains compared with the vulture-derived isolate at the nucleotide level. The maximum-likelihood tree was inferred using IQ-TREE v3.0.1 under the GTR+F+G4 substitution model, with branch support shown in **(A)**. Coding regions were individually extracted from complete genome sequences and aligned to calculate pairwise identities, visualized as a heatmap.

This study reports the 11th coding-complete genome of AOAV-16 deposited in public databases (GenBank accession no. PX482736) and the first from a non-*Anseriformes* host. Using high-throughput next-generation sequencing (NGS), we generated a 15,139-nucleotide coding-complete genome for AOAV-16/cinereous_vulture/South_Korea/23WF447-15P/2023 (vulture-derived AOAV-16) with a mean sequencing depth of 17,151 × . The length of the complete CDS was 14,904 nt, consistent with the paramyxovirus “rule of six” (18). Metagenomic classification using CZID identified reads mapping to avulaviruses, thereby supporting AOAV-16 as the predominant viral species in the sample and guiding selection of the reference genome for assembly. The genome exhibited the canonical avulavirus gene order 3'-NP-P-M-F-HN-L5'), and the F protein cleavage site was monobasic (^110^LVQAR↓L^115^), a motif conserved across all known AOAV-16 isolates and indicative of low virulence (Figure 1B). Notably, the HN-to-L intergenic region contained a 12-nt deletion in the hexanucleotide repeat array (AAAAAU)n compared to other AOAV-16 strains, as confirmed by raw read mapping (8). Mitochondrial DNA barcoding confirmed the host species of the fecal sample as a cinereous vulture.

**Figure 2:**
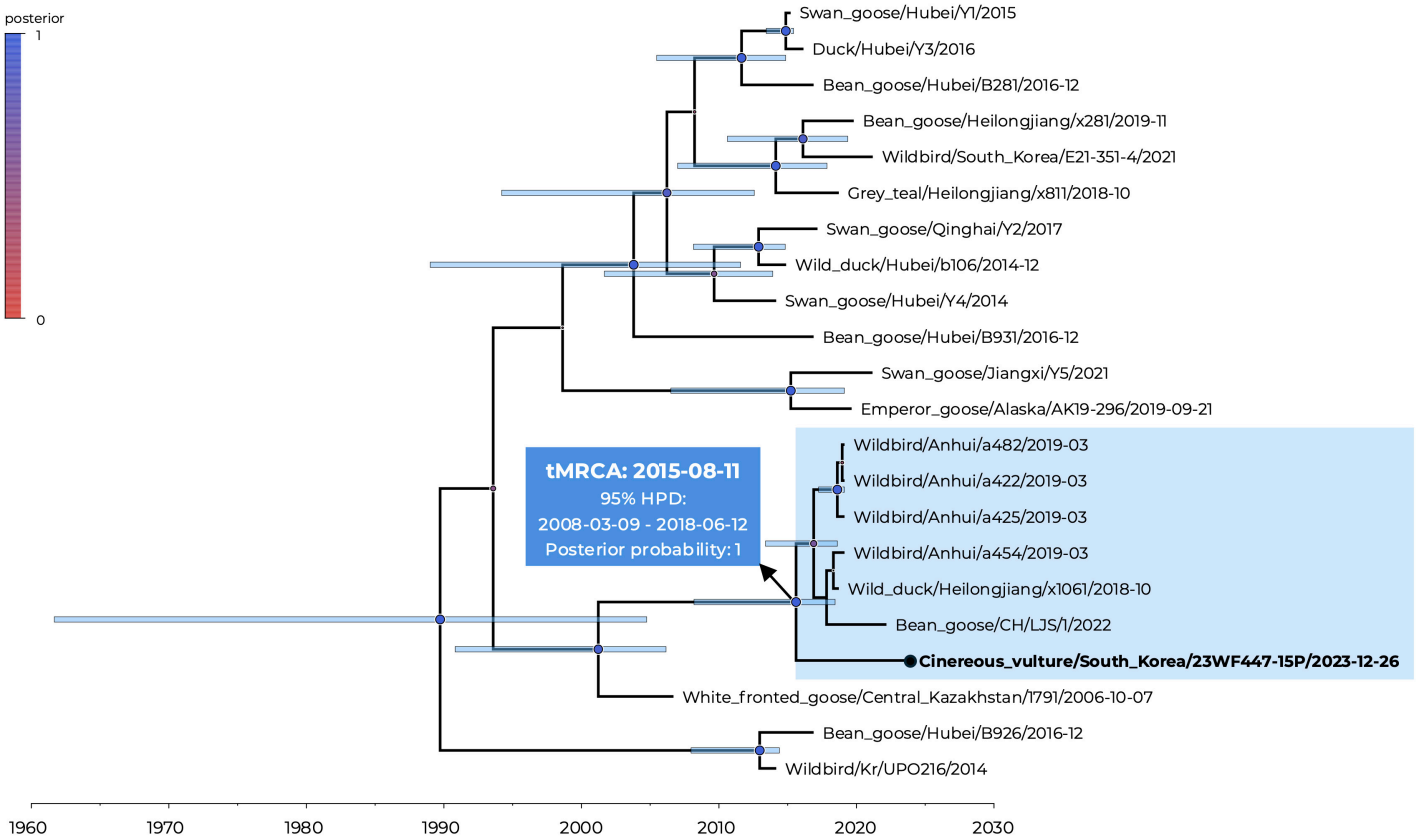
Temporal phylogenetic analysis of Avian orthoavulavirus 16 (AOAV-16). A time-scaled maximum clade credibility tree was inferred from complete F gene sequences using BEAST v10.5.0 under the TN93+F+I substitution model, an uncorrelated lognormal relaxed clock, and a GMRF Bayesian Skyride coalescent prior. Node size and color indicate posterior probability support. Node bars indicate the 95% highest posterior density (HPD) interval of the node height. The vulture-derived AOAV-16 sequence generated in this study is shown in bold and marked with a red circle. The blue box denotes the estimated time to the most recent common ancestor (tMRCA), along with the 95% HPD interval and posterior probability of the divergence node associated with the cinereous vulture isolate.

Phylogenetic analysis confirmed that the vulture-derived isolate clustered monophyletically within the AOAV-16 clade, which is most closely related to AOAV-1 at the species level (Figure 1A). Pairwise nucleotide distance analysis revealed the highest nucleotide sequence identities to viruses identified from China (Bean_goose/CH/LJS/1/2022; 97.2% identity) and Kazakhstan (White_fronted_goose/Central_Kazakhstan/1791/2006; 96.8% identity), with the M gene exhibiting the greatest divergence among all coding sequences (Figure 1C, Supplementary Table 1). Time-scaled Bayesian phylogenetic analysis indicated that the vulture-derived isolate shared a common ancestry with Chinese waterfowl origin viruses that potentially diverged since approximately August 2015 (95% highest posterior density [HPD]: March 2008 to June 2018) with high posterior probability support (Figure 2). However, the tMRCA of the root was inferred to be 22 September 1989, with a wide 95% Bayesian credible interval (4 September 1961 to 30 September 2004), reflecting limited sequence data availability. The mean substitution rate was 7.66 × 10^−4^ substitutions/site/year (s/s/y, 95% HPD: 1.84 × 10^−4^−1.52 × 10^−3^). Nucleotide divergence among available AOAV-16 genomes ranged from 0.14% to 3.2% despite a decade-long temporal and geographic span, a pattern that may be consistent with limited selective pressure in wild hosts. The geographic clustering of all AOAV-16 viruses within the East Asian–Australasian Flyway (EAAF) suggests sustained transmission along this migratory corridor. However, virus propagation in embryonated chicken eggs prior to sequencing may have introduced selection bias, potentially favoring egg-adapted variants over the original fecal viral population (19).

The isolation of AOAV-16 from fecal droppings of a cinereous vulture—a scavenging raptor in the order *Accipitriformes*—represents the first detection in a non-*Anseriformes* host. This finding raises questions regarding the ecological contexts in which AOAV-16 genomic RNA may be detected beyond aquatic birds, including potential exposure scenarios associated with scavenging behavior. Importantly, fecal detection of viral RNA does not necessarily indicate productive infection (20), whereas low-level fecal or cloacal RNA detection has been associated with environmental contamination and potential cryptic circulation (21).

Taken together, the present findings warrant cautious interpretation but suggest that including non-traditional avian species—particularly scavengers such as cinereous vultures—may help capture a broader range of viral detection contexts. Further longitudinal, multi-host studies in shared habitats will be needed to clarify their relevance to transmission dynamics and poultry spillover risk.

## Data Availability

The datasets presented in this study can be found in online repositories. The names of the repository/repositories and accession number(s) can be found in the article/Supplementary material.

## Supplementary material

Supplementary Figure 1 Maximum-likelihood phylogeny based on the L protein amino acid sequences of representative avian orthoavulaviruses.

Supplementary Table 1 Variation in HN-L intergenic repeat patterns among AOAV-16 isolates.

Supplementary Table 2 Pairwise nucleotide identity between cinereous_vulture/South_Korea/23WF447-15P/2023 and other AOAV-16 strains.

Supplementary Table 3 Genome organization of AOAV-16 cinereous_vulture/South_Korea/23WF447-15P/2023.
