# Supplementary material for: Isolation and whole genome sequencing of an Avian orthoavulavirus 16 from cinereous vulture (Aegypius monachus) in South Korea, 2023
Source: Front Vet Sci. 2026 Feb 6;13:1739779. doi: 10.3389/fvets.2026.1739779 (PMC12921703; doi:10.3389/fvets.2026.1739779)
Supplement: Supplementary Figure 1 — Maximum-likelihood phylogeny based on the L protein amino acid sequences of representative avian orthoavulaviruses. [file Data_Sheet_1.docx]

**
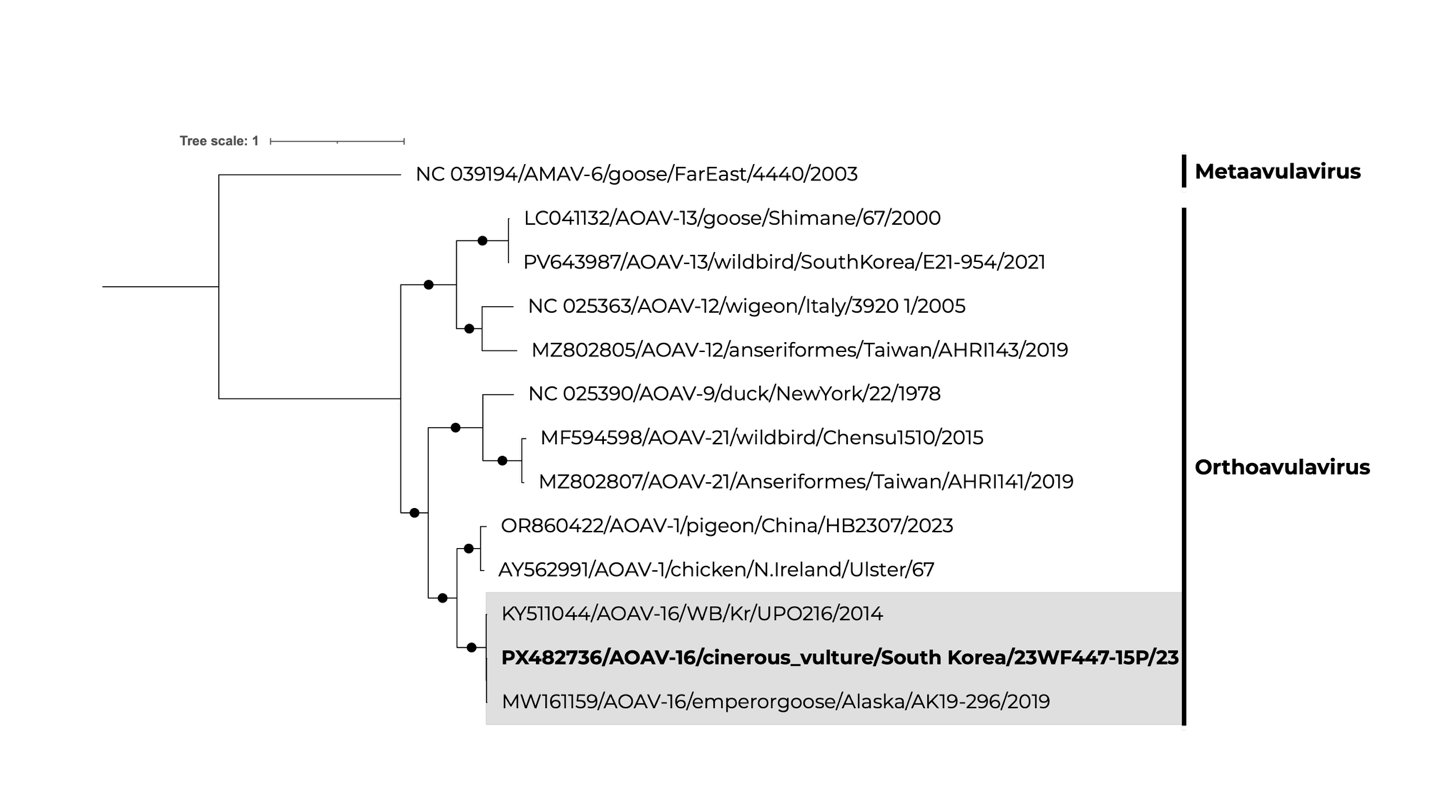
**

**Supplementary Figure 1.** Maximum-likelihood phylogeny based on the L protein amino acid sequences of representative avian orthoavulaviruses. The phylogenetic tree was inferred using IQ-TREE v3.0.1 under the Q.YEAST+F+I+R3 substitution model, with branch support assessed by 1,000 ultrafast bootstrap replicates and 1,000 SH-aLRT replicates. The resulting tree was visualized using iTOL. Avian metaavulavirus 6 (NC_039194/AMAV-6/goose/FarEast/4440/2003) was included as an outgroup. The isolate generated in this study is indicated in bold.

**Supplementary Table 1.** Variation in HN–L intergenic repeat patterns among AOAV-16 isolates

| Virus | HN-L intergenic repeat pattern | Net deletion (nt) |
| --- | --- | --- |
| wild_bird/South_Korea/UPO216/2014 | ------------------ | 18 |
| white_fronted_goose/Central_Kazakhstan/1791/2006 | AAAAAUAAAAAUAAAAAU | 0 |
| bean_goose/CH/LJS/1/22 | ------AAAAAUAAAAAU | 6 |
| Cinereous Vulture/South Korea/23WF447-15P/2023 | ------------AAAAAU | 12 |
| swan_goose/Jiangxi/Y5/2021 | AAAAAUAAAAAUAAAAAU | 0 |
| emperor_goose/Alaska/AK19-296/2019 | AAAAAUAAAAAUAAAAAU | 0 |
| swan_goose/Hubei/Y4/2014 | ------AAAAAUAAAAAU | 6 |
| swan_goose/Qinghai/Y2/2017 | ------AAAAAUAAAAAU | 6 |
| wild_bird/South_Korea/E21-351-4/2021 | ------------AAAAAU | 12 |
| swan_goose/Hubei/Y1/2015 | ------------AAAAAU | 12 |
| duck/Hubei/Y3/2016 | AAAAAUAAAAAUAAAAAU | 0 |

**Supplementary Table 2.** Pairwise nucleotide identity between cinereous_vulture/South_Korea/23WF447-15P/2023 and other AOAV-16 strains.

| Virus | Pairwise nucleotide identity against cinereous_vulture/South_Korea/23WF447-15P/2023 (%) ^a^ | | | | | | |
| --- | --- | --- | --- | --- | --- | --- | --- |
|  | Complete Genome | NP | P | M | F | HN | L |
| wild_bird/South_Korea/UPO216/2014 | 96.8 | 98.17 | 97.53 | 95.83 | 97.16 | 96.39 | 97.37 |
| white_fronted_goose/Central_Kazakhstan/1791/2006 | 98.34 | 99.05 | 98.9 | 98.08 | 99.09 | 97.47 | 98.8 |
| bean_goose/CH/LJS/1/22 | 99.29 | 99.73 | 99.36 | 99.42 | 99.52 | 99.3 | 99.56 |
| swan_goose/Jiangxi/Y5/2021 | 97.43 | 98.31 | 97.81 | 96.46 | 98.55 | 97.23 | 98.17 |
| emperor_goose/Alaska/AK19-296/2019 | 97.46 | 98.24 | 97.9 | 96.67 | 98.19 | 97.09 | 98.24 |
| swan_goose/Hubei/Y4/2014 | 97.68 | 98.04 | 97.99 | 97.17 | 98.61 | 97.25 | 98.34 |
| swan_goose/Qinghai/Y2/2017 | 97.63 | 97.97 | 97.9 | 97.25 | 98.37 | 97.31 | 98.32 |
| wild_bird/South_Korea/E21-351-4/2021 | 97.34 | 97.9 | 97.44 | 97.08 | 97.89 | 97.04 | 97.97 |
| swan_goose/Hubei/Y1/2015 | 97.79 | 98.51 | 98.17 | 96.92 | 98.25 | 97.42 | 98.41 |
| duck/Hubei/Y3/2016 | 97.69 | 98.51 | 98.17 | 96.92 | 98.25 | 97.42 | 98.35 |

^a^ NP, nucleoprotein; P, phosphoprotein; M, matrix protein; F, fusion protein; HN, hemagglutinin-neuraminidase; L, large polymerase protein.

**Supplementary Table 3.** Genome organization of AOAV-16 cinereous_vulture/South_Korea/23WF447-15P/2023.

|  | Region (length, nt) | | Sequence^b^ | |
| --- | --- | --- | --- | --- |
|  | Gene | CDS^a^ | Gene start | Gene end |
| Leader | -32 (32) |  |  |  |
| N | 33-1790 (1758) | 99-1574 (1476) | ACGGGTAGAA | TTAGAAAAAA |
| P | 1793-3267 (1475) | 1876-3075 (1200) | ACGGGTAGAA | TTAAGAAAAAA |
| M | 3269-4510 (1242) | 3303-4397 (1095) | ACGGGTAGAA | TTACAAAAAAA |
| F | 4517-6339 (1823) | 4569-6224 (1656) | ACGGGGAGAA | TTAGAAAAAA |
| HN | 6340-8362 (2023) | 6431-8287 (1857) | ACGGGGAGAA | TTAGAAAAAA |
| L | 8383-15092 (6710) | 8394-15002 (6609) | ACGGGGAGAA | TTAAGAAAAAA |
| Trailer | 15093-15139 (47) |  |  |  |

^a^ CDS lengths are shown in parentheses

^b^ Conserved gene start and gene end motifs are indicated for each gene.
